# Diversity of the immune microenvironment and response to checkpoint inhibitor immunotherapy in mucosal melanoma

**DOI:** 10.1172/jci.insight.179982

**Published:** 2024-11-08

**Authors:** Joris L. Vos, Joleen J.H. Traets, Xiaohang Qiao, Iris M. Seignette, Dennis Peters, Michel W.J.M. Wouters, Erik Hooijberg, Annegien Broeks, Jacqueline E. van der Wal, M. Baris Karakullukcu, W. Martin C. Klop, Arash Navran, Marc van Beurden, Oscar R. Brouwer, Luc G.T. Morris, Mariette I.E. van Poelgeest, Ellen Kapiteijn, John B.A.G. Haanen, Christian U. Blank, Charlotte L. Zuur

**Affiliations:** 1 Department of Head and Neck Surgery and Oncology and; 2 Division of Tumor Biology and Immunology, Netherlands Cancer Institute, Amsterdam, Netherlands.; 3 Head and Neck Service, Immunogenomic Oncology Platform, Department of Surgery, Memorial Sloan Kettering Cancer Center, New York, New York, USA.; 4 Division of Molecular Oncology and Immunology,; 5 Department of Pathology,; 6 Core Facility Molecular Pathology and Biobanking, and; 7 Department of Surgery, Netherlands Cancer Institute, Amsterdam, Netherlands.; 8 Department of Oral and Maxillofacial Surgery, Amsterdam University Medical Center – Location Amsterdam Medical Center, Amsterdam, Netherlands.; 9 Department of Radiation Oncology,; 10 Department of Gynecology, and; 11 Department of Urology, Netherlands Cancer Institute, Amsterdam, Netherlands.; 12 Department of Gynecology and; 13 Department of Medical Oncology, Leiden University Medical Center, Leiden, Netherlands.; 14 Department of Medical Oncology, Netherlands Cancer Institute, Amsterdam, Netherlands.; 15 Department of Otorhinolaryngology, Leiden University Medical Center, Leiden, Netherlands.

**Keywords:** Immunology, Oncology, Cancer immunotherapy, Melanoma, Molecular biology

## Abstract

Mucosal melanoma (MucM) is a rare cancer with a poor prognosis and low response rate to immune checkpoint inhibition (ICI) compared with cutaneous melanoma (CM). To explore the immune microenvironment and potential drivers of MucM’s relative resistance to ICI drugs, we characterized 101 MucM tumors (43 head and neck [H&N], 31 female urogenital, 13 male urogenital, 11 anorectal, and 3 other gastrointestinal) using bulk RNA-Seq and immunofluorescence. RNA-Seq data show that MucM has a significantly lower IFN-γ signature levels than CM. MucM tumors of the H&N region show a significantly greater abundance of CD8^+^ T cells, cytotoxic cells, and higher IFN-γ signature levels than MucM from lower body sites. In the subcohort of 35 patients with MucM treated with ICI, hierarchical clustering reveals clusters with a high and low degree of immune infiltration, with a differential ICI response rate. Immune-associated gene sets were enriched in responders. Signatures associated with cancer-associated fibroblasts, macrophages, and TGF-β signaling may be higher in immune-infiltrated, but ICI-unresponsive tumors, suggesting a role for these resistance mechanisms in MucM. Our data show organ region–specific differences in immune infiltration and IFN-γ signature levels in MucM, with H&N MucM displaying the most favorable immune profile. Our study might offer a starting point for developing more personalized treatment strategies for this disease.

## Abstract 2

This multi-modality analysis provides insights into the mucosal melanoma microenvironment, providing a starting point for developing more personalized immunotherapy for patients with this rare and treatment-resistant cancer.

## Introduction

Mucosal melanoma (MucM) is a rare subtype of malignant melanoma that accounts for 1.3% of all melanomas in the US (1); it has an incidence of 1.5–2.8 per million person-years in The Netherlands (2). In Asian populations, however, it constitutes up to 23% of melanomas (3). The head and neck (H&N) (55%), anorectal (24%), and urogenital (18%) regions are most affected, yet MucM may arise in any mucosal surface (1). MucM has often progressed substantially before diagnosis: 18%–40% of patients present with regional lymph node metastases and 14%–23% with distant metastases (1, 4, 5). Curative treatment for primary MucM consists of wide local excision of the primary tumor with lymph node dissection in case of nodal involvement (6). Postoperative radiotherapy (RT) is often administered to improve locoregional control (7). Still, the 5-year overall survival (OS) for patients with MucM is only 25%‒32% (1, 8). In the case of distant metastases, MucM is incurable.

Treatment with immune checkpoint inhibitors (ICIs) directed against PD-1 as monotherapy or combined with anti–CTLA-4 ICI has durably improved outcomes for patients with stage IV CM (9–11). However, patients with metastatic MucM are less likely to benefit from (dual) ICI than metastatic CM (12–14). Neoadjuvant treatment with anti–PD-1 monotherapy or combined anti–PD-1 and anti–CTLA-4 drugs, however, has been observed to induce a pathologic response in 35% of patients with resectable MucM, who had a significantly superior event-free survival compared with nonresponding patients — highlighting the promise of neoadjuvant ICI in this rare cancer (15).

Molecular investigations have shown that MucM is characterized by a mutational and copy number profile that differs from that of CM — such as a lower tumor mutational burden (TMB; 2.3–2.7 vs. 36.3–49.2 mutations/Mb, respectively) (16–18), which likely contributes to MucM’s relatively poor response rate to ICI compared with CM. However, tumor immune microenvironmental aspects important to ICI response (19), such as the abundance of tumor-infiltrating lymphocytes (20) and the presence of an IFN-γ gene expression signature (21–23), have not been extensively studied in MucM (24–27).

To address this gap in knowledge, we assembled a cohort of 101 patients with H&N, female and male urogenital, anorectal, or gastrointestinal mucosa MucM treated at the Netherlands Cancer Institute (NKI) and Leiden University Medical Center (LUMC) between 1997 and 2020. Thirty-five patients received any form of ICI as part of treatment for metastatic MucM. We performed bulk mRNA sequencing (RNA-Seq) and multiplex immunofluorescence (mIF) staining to investigate the presence of immune cell populations and inflammation in the MucM microenvironment at different primary sites. In addition, the MucM immune microenvironment was compared with a dataset previously generated in patients who had received neoadjuvant ICI for stage III CM (21). Our multimodality analysis provides insights into the MucM microenvironment and offers a step toward more rational immunotherapy for patients with this rare and treatment-resistant malignancy.

## Results

### Patients with primary MucM experience poor survival.

**Figure 1:**
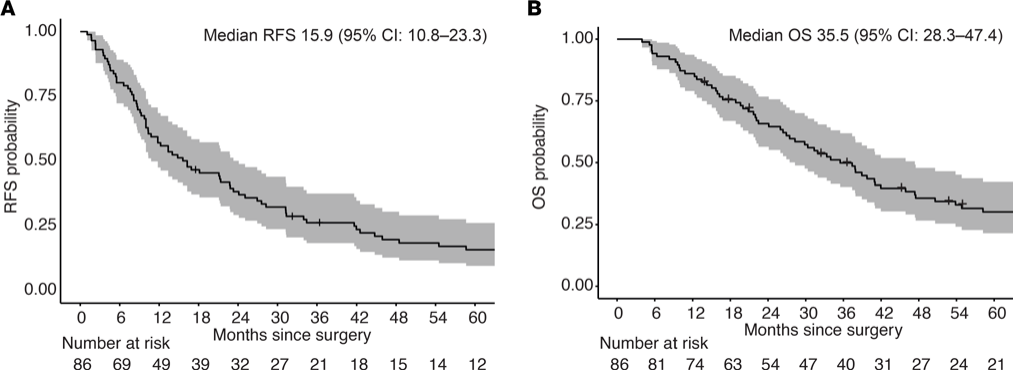
Relapse-free and overall survival since surgery of all 86 patients with mucosal melanoma treated with surgery. (**A**) Kaplan-Meier estimates for RFS since surgery with 95% CIs. (**B**) Kaplan-Meier estimates for OS since surgery with 95% CIs. RFS, relapse-free survival; OS, overall survival; MucM, mucosal melanoma; H&N, head and neck.

One hundred and one patients (61 female and 40 male individuals; median age, 69 years) whose FFPE tumor material was available were treated for MucM between January 1997 and December 2019 (Table 1), including 43 patients (42.6%) with MucM of the H&N region, of whom 40 had sinonasal and 3 had oral cavity MucM. Fifty-eight patients (57.4%) had MucM of lower body regions: 31 had MucM of the female urogenital tract (FUT), 13 had MucM of the male urogenital tract (MUT), 11 had MucM of the anorectal (AR) reigon, and 3 had MucM gastrointestinal (GI) MucMs. Eighty-six patients (85%) were initially treated with surgery (35 received adjuvant RT), and 15 patients (15%) received only palliative local and/or systemic treatments after diagnosis. Median relapse-free survival and OS for the 86 patients with MucM treated with surgery (with or without adjuvant RT) were 15.9 months (95% CI, 10.8‒23.3 months) and 35.5 months (95% CI, 28.3‒47.4 months), respectively (Figure 1, A and B).

### Response to immune checkpoint inhibition is associated with OS.

Thirty-five patients (11 sinonasal, 14 FUT, 7 AR, 2 MUT, 1 GI) received any form of ICI (Table 2) for irresectable stage III or stage IV MucM at primary diagnosis (*n* = 4) or after initial surgery (*n* = 31). Patients received combined anti–CTLA-4 + anti–PD-1 therapy (*n* = 14, 40%), anti–PD-1 monotherapy (*n* = 10, 29%), or anti–CTLA-4 monotherapy (*n* = 11, 31%) as the first line of ICI. Four of the 11 patients who were first treated with anti–CTLA-4 monotherapy went on to receive anti–PD-1 upon progression. Eight patients had received one or more lines of systemic therapy before ICI was started (5 chemotherapy, 2 targeted therapy, and 1 adjuvant anti–PD-1 after surgery + postoperative RT).

An objective response (OR) based on imaging (23 CT, 10 ^18^F-FDG-PET/CT [FDG-PET]) was observed in 10/35 patients (28.6%, Table 3), including 6 patients with a complete response (CRs). Three patients (8.6%) had stable disease (SD) as their best response, while 22 patients (62.9%) developed progressive disease (PD), including 2 patients (1 sinonasal and 1 FUT MucM) with symptomatic disease progression who died before first response imaging (38 and 39 days after ICI start). The OR rate (ORR) was higher in patients with H&N (4 of 11, 36.4%) than lower-region MucM (6 of 24, 25%), but this difference was not statistically significant (*P* = 0.69).

**Figure 2:**
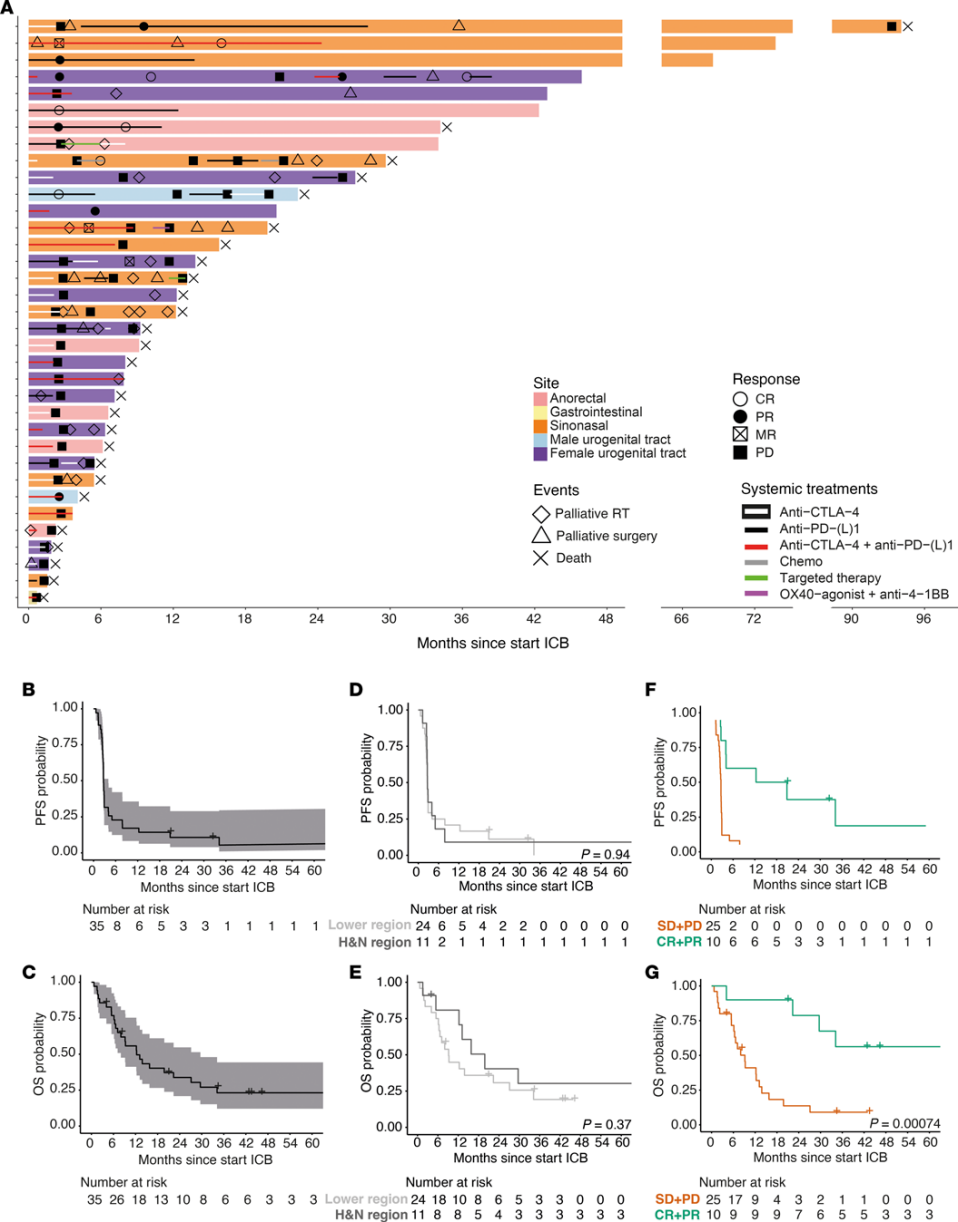
Swimmer plot, progression-free survival, and overall survival of the subcohort of 35 patients with MucM treated with ICB. (**A**) Swimmer plot visualizing each patient treated with ICI as an individual bar. All bars begin at the point at which a patient first started ICI therapy and end at the last follow-up date; deaths from any cause are marked with an X. Bar colors indicate primary MucM location. Lines within bars indicate systemic palliative treatments, from the first to the last administered dose. Palliative radiotherapy and surgical interventions are marked with diamonds and triangles, respectively. CR, PR, and PD are marked with the symbols displayed in the legend. Patients with progressive disease in the context of a mixed response (MR) are indicated. The 2 patients with rapid symptomatic PD, in whom no imaging response assessment was performed, are included with the date of clinical progression as PD date. Please note that the *x* axis is interrupted twice. (**B**) Kaplan-Meier estimates and 95% CI for PFS since the start of the first ICI therapy for the whole ICI subcohort. (**C**) OS with 95% CI since the start of ICI therapy for the whole cohort. (**D**) PFS since the start of ICI, stratified per primary MucM region. *P* values were calculated using a 2-sided log-rank test. (**E**) OS since the start of ICI, stratified per primary MucM region. *P* values were calculated using a 2-sided log-rank test. (**F**) PFS since the start of ICI, stratified per best objective response. *P* values were calculated using a 2-sided log-rank test. (**G**) OS since the start of ICI, stratified per best objective response. The number at risk refers to the total number of patients who have not yet experienced the event of interest or been censored at the specified time points *P* values were calculated using a 2-sided log-rank test. RT, radiotherapy; PFS, progression-free survival; OS, overall survival; MucM, mucosal melanoma; H&N, head and neck; SN, sinonasal; OC, oral cavity, FUT, female urogenital tract; MUT, male urogenital tract; AR, anorectal; GI, gastrointestinal; CR, complete response; PR, partial response; SD, stable disease; PD, progressive disease; MR, mixed response.

A swimmer plot overview of individual patient systemic treatments, palliative local treatments, and responses is shown in Figure 2A. Median PFS and OS were 2.7 months (95% CI, 2.6‒4.0 months) and 12.3 months (95% CI, 8.0‒29.months) since ICI treatment initiation, respectively (Figure 2, B and C). Median PFS and OS were not significantly different between H&N and lower region MucM (2.7 months [95% CI, 2.6‒NA months] vs. 2.7 months [95% CI, 2.5‒4.1 months], *P* = 0.94; and 19.8 months [95% CI, 12.2‒NA months] vs. 9.2 months [95% CI, 6.6‒34.2 months], *P* = 0.37, respectively; Figure 2, D and E). PFS and OS were significantly superior in patients responding to ICI (median, 16.6 months [95% CI, 4.0‒NA months] and 94.1 months [95% CI, 29.6‒NA months], respectively) compared with nonresponding patients (median, 2.7 months [95% CI, 2.4‒2.9 months] and 9.2 months [95% CI, 6.3‒13.8 months], *P* = 0.0002 and 0.0007, respectively; Figure 2, F and G).

### Characterization of the immune microenvironment suggests low immune infiltration in MucM compared with cutaneous melanoma.

**Figure 3:**
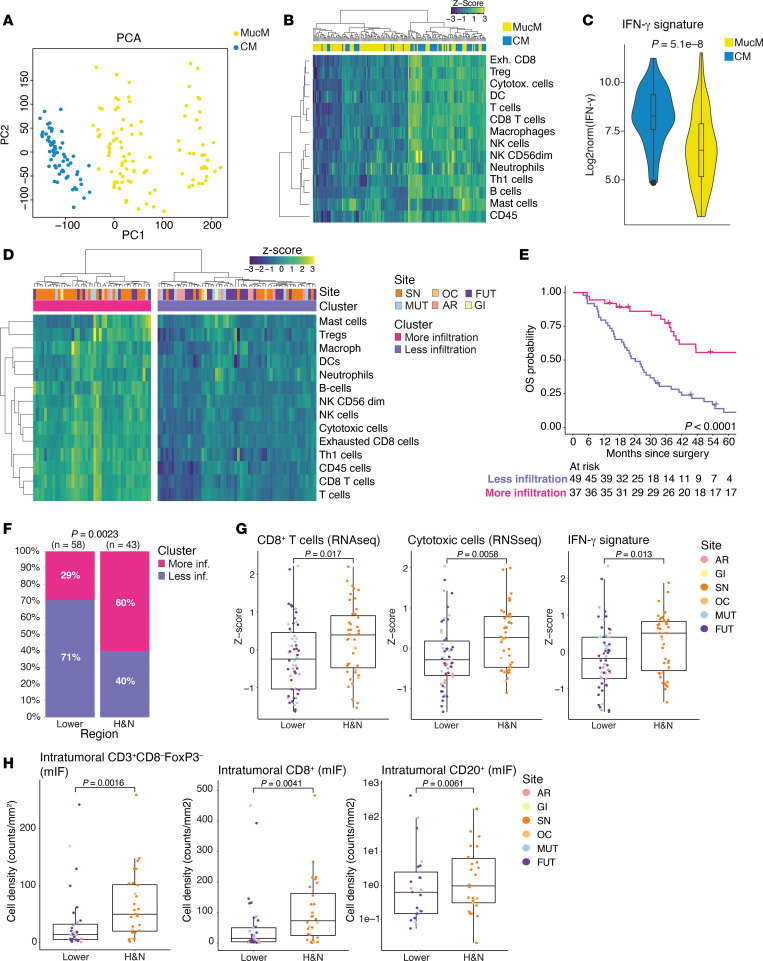
RNA-Seq– and mIF-based characterization of the MucM immune microenvironment across primary sites and compared with CM dataset (21). (**A**) PCA of primary MucM (*N* = 101) and CM (*N* = 64) samples after computational batch correction. (**B**) Hierarchical clustering heatmap of CM and MucM samples per the Danaher (30) immune cell signatures. (**C**) Box-and-violin plots visualizing the presence of the 10-gene IFN-γ RNA signature (23) in batch-corrected MucM vs. CM samples. (**D**) Heatmap leukocyte gene expression signatures in MucM, with hierarchical clustering divides samples into less-infiltrated (violet) and more-infiltrated (magenta) groups. Samples are further annotated with their primary MucM site of origin. (**E**) Kaplan-Meier overall survival estimate in all patients treated with surgery (*N* = 86), stratified by immune cluster identified in **D**. Exact *P* values were calculated using a 2-sided log-rank test. (**F**) Stacked bar plot showing the fraction of lower and H&N region MucM clustering as either more or less infiltrated. The *P* value was calculated using Fisher’s exact test. (**G**) Box plots showing the *Z*-scores of CD8^+^ T cell, cytotoxic cell, and Ayers’ 10-gene IFN-γ signature across MucM regions. (**H**) Box plots showing the density of CD3^+^CD8^–^FoxP3^–^, CD8^+^, and CD20^+^ cells located within the tumor parenchyma, assessed through digital analysis of mIF-stained slides. The CD20^+^ plot’s *y* axis was log_10_-transformed. Dot colors in **G** and **H** correspond to the MucM site. Box plots in **C**, **G**, and **H** show medians, IQRs, and whiskers up to 1.5 times the IQR. The violin in **C** displays the probability density of the data. *P* values in **C** and **G**, and **H** were calculated using a 2-sided Wilcoxon’s rank-sum test. MucM, mucosal melanoma; CM, cutaneous melanoma; H&N, head and neck; SN, sinonasal; OC, oral cavity, FUT, female urogenital tract; MUT, male urogenital tract; AR, anorectal; GI, gastrointestinal; PCA, principal component analysis.

We performed batch correction (28) and subsequently compared the MucM transcriptome to the OpACIN-neo (21, 29) bulk RNA-Seq datasets, previously generated from pretreatment biopsies of lymph nodal metastases of 64 patients with stage III CM treated with neoadjuvant nivolumab (anti–PD-1) and ipilimumab (anti–CTLA-4). Principal component analysis revealed separation of MucM and CM samples (Figure 3A), highlighting the distinct transcriptomes of these tumors. Profiling of the tumor immune microenvironment using the gene expression signatures developed by Danaher et al. (30) showed clustering of strongly immune infiltrated (mostly CM) and less immune infiltrated (mostly MucM) samples (Figure 3C) and significantly higher IFN-γ signature (23) levels in CM as compared with MucM tumors (*P* = 5.1 × 10^–8^, Figure 3C). These results remained significant when analyses were repeated using DESeq (31) normalization of batch-uncorrected datasets (Supplemental Figure 1, A‒C; supplemental material available online with this article; https://doi.org/10.1172/jci.insight.179982DS1), and suggest that MucM is characterized by a lower immune cell infiltration and IFN-γ signature than CM.

### Immune infiltration at the H&N site is higher than other MucM sites of primary origin.

To explore differences in the immune microenvironment of MucM at different mucosal regions of origin, we used RNA-Seq data to analyze immune infiltration within the MucM cohort specifically. Upon hierarchical clustering, we identified a cluster with more (*n* = 43) and one with less immune cell infiltration (*n* = 58; Figure 3D). As expected, the IFN-γ signature levels were significantly higher in the more-infiltrated cluster compared with the less-infiltrated cluster (*P* = 6.5 × 10^–16^, Supplemental Figure 1D). OS from date of surgery for the 86 patients with MucM treated with curative intent was significantly superior in the more-infiltrated cluster compared with that in the less-infiltrated cluster (median, 71.9 months [95% CI, 41.0–NA months] and 24.1 months [95% CI, 19.6–31.9 months], respectively; *P* < 0.0001; Figure 3E). Interestingly, H&N MucM was significantly overrepresented in the more-infiltrated cluster, with 60% of H&N MucM categorized as infiltrated compared with only 29% of lower body region MucMs (*P* = 0.0023, Figure 3F). Analysis per site suggested that AR and FUT MucM are particularly less-infiltrated tumors (18% and 26% classified as infiltrated, respectively; Supplemental Figure 1E). H&N MucMs were characterized by a significantly higher CD8^+^ T cell, cytotoxic cell, and IFN-γ signature compared with other, lower body region MucMs (*P* = 0.02, *P* = 0.01, and *P* = 0.01, respectively; Figure 3G). Expression of other immune cell populations in H&N and lower body MucM are visualized in Supplemental Figure 1F.

To enumerate the immune cells within the MucM microenvironment, whole slides from 64 patients (25 sinonasal, 3 oral cavity, 21 FUT, 9 AR, and 6 MUT) were stained with a mIF panel for CD3, CD8, CD20, CD68, and FoxP3. Automated cell segmentation (Supplemental Figure 2) revealed that, as expected, the total CD8^+^ T cell density (intratumoral and stromal combined, in cells/mm^2^) was strongly and significantly correlated with Danaher’s CD8^+^ T cell, overall T cell, and cytotoxic T cell RNA signatures (respective Rho = 0.82, 0.79, and 0.77; respective *P* = 0.0 × 10^0^, 8.9 × 10^–15^, and 8.0 × 10^–15^, Supplemental Figure 3A). The total CD20^+^ cell density, too, was strongly and significantly correlated with the B cell RNA signature (Rho = 0.70, *P* = 1.4 × 10^–10^). The correlations between the CD68^+^ cell density and the macrophage RNA signature, and the FoxP3^+^ cell density and the Treg RNA signatures, were weaker but still statistically significant (Rho = 0.37 and Rho = 0.54; *P* = 0.02 and *P* = 4.1 × 10^–6^, respectively). The densities of the stained cell populations per MucM organ site are summarized in Supplemental Table 1. There was no significant difference in immune cell density in the stromal compartment for any of the investigated markers between the MucM regions (Supplemental Figure 3B). However, the density of intratumorally located CD3^+^CD8^–^FoxP3^–^, CD8^+^, and CD20^+^ cells was significantly higher in H&N MucM (49.3, 72.2, and 0.7 cells/mm^2^, respectively) compared with lower region MucM (13.5, 15.3, and 0.1 cells/mm^2^, respectively; *P* = 0.002, *P* = 0.004, *P* = 0.006, respectively, Figure 3H). There were no statistically significant differences in the intratumoral density of FoxP3^+^ or CD68^+^ cells between H&N and lower region MucM (Supplemental Figure 3C). Finally, the median ratios of the intratumoral to stromal cell densities for CD3^+^CD8^–^FoxP3^–^, CD20^+^, and CD8^+^ cells were higher in H&N compared with lower region MucM (*P* = 0.04, *P* = 0.03, and *P* = 0.06 respectively; Supplemental Figure 3D).

In summary, our comprehensive immune characterization based on RNA-Seq and mIF data shows that intratumoral immune cell infiltration, including T cell populations, is positively associated with OS in MucM. Furthermore, primary H&N MucM is more densely infiltrated in this MucM cohort than MucM from the lower body mucosa, including the FUT, MUT, and AR region.

### ICI-responding MucM tumors may have a higher CD8^+^ T cell and IFN-γ signature before treatment.

**Figure 4:**
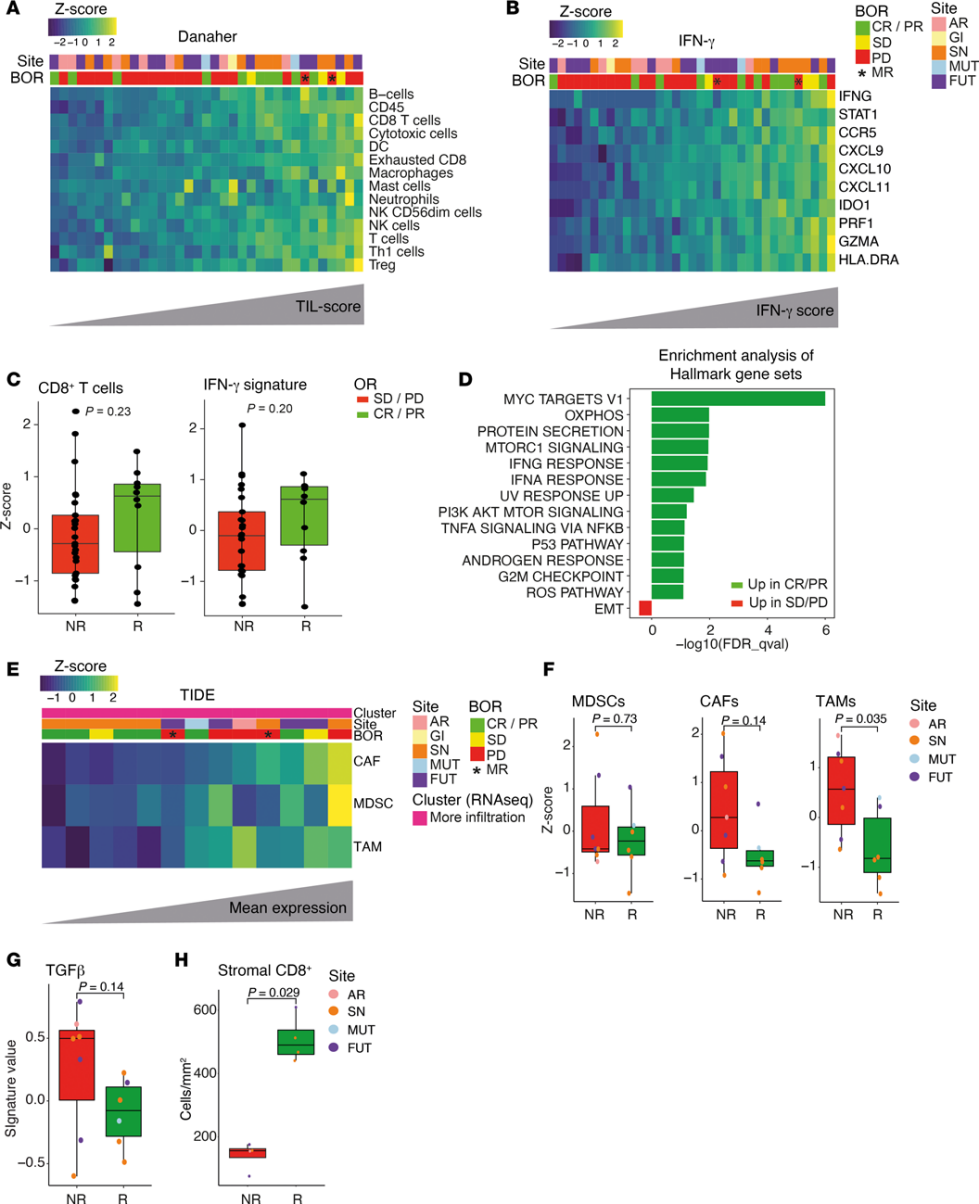
Microenvironmental correlates of response to ICI in mucosal melanoma. (**A**) Heatmap of samples’ Danaher leukocyte RNA signatures, ordered by the mean across all signatures (the TIL-score). (**B**) Heatmap of genes associated with IFN-γ signaling (23), ordered by mean expression the IFN-γ signature. (**C**) Box plots of *Z*-scores of CD8^+^ T cell and IFN-γ signatures per ICI response category (responders, green; nonresponders, red). (**D**) Enrichment analysis of Hallmark gene sets (32) in responders and nonresponders, ordered by FDR. (**E**) Heatmap visualizing TIDE signatures (33) associated with CAFs, MDSCs, and TAMs in all patients from the more-infiltrated cluster defined in Figure 3D. Heatmap is organized by the mean across the 3 signatures. (**F**) Box plots of TIDE signature values in responders vs. nonresponders from the more-infiltrated cluster. (**G**) Box plots displaying the TGF-β signatures in responders vs. nonresponders from the more-infiltrated cluster. (**H**) Box plot visualizing the mIF-assessed stromal CD8^+^ T cell density in responding vs. nonresponding samples from the more-infiltrated cluster. Tracks in **A**, **B**, and **E** annotate sample’s primary site of origin and best objective response, with asterisks indicating tumprs that progressed in the context ofa mixed response. Boxes in **C** and **F**–**H** indicate the median and IQR; with whiskers extending up to 1.5 times the IQR. Exact *P* values in **C** and **F**–**H** were calculated using a Wilcoxon’s rank-sum test. Dot colors represent samples’ site of primary MucM origin. H&N, head and neck; SN, sinonasal; OC, oral cavity, FUT, female urogenital tract; MUT, male urogenital tract; AR, anorectal; GI, gastrointestinal; BOR, best objective response; NR, no response; R, response; CR, complete response; PR, partial response; SD, stable disease; PD, progressive disease; MR, mixed response; TIDE, tumor immune dysfunction and exclusion; MDSC, myeloid-derived suppressor cell; CAF, cancer-associated fibroblast; TAM, tumor-associated macrophage; APM, antigen-presenting machinery.

We compared the 10 responders (CR or partial response [PR]) with the 25 nonresponding patients (SD or PD) in the ICI-treated subcohort to elucidate potential biomarkers for ICI response in MucM. Of note, 2 samples from patients with PD (1 sinonasal and 1 FUT) as the best response were obtained from lesions that regressed after ICI as part of a mixed response. The tumor samples from which RNA was isolated were obtained a median of 284 days (IQR, 80–599 days) prior to the start of ICI treatment. The CD8^+^ T cell and IFN-γ signatures were higher in responders compared with nonresponders (median *Z*-scores 0.63 versus –0.29, and 0.61 vs. –0.10, respectively) but not significant (*P* = 0.23 and *P* = 0.20, Figure 4, A‒C). Similarly, the median mIF-assessed intratumoral and stromal CD8^+^ T cell density, available for 25 of the 35 ICI-treated patients, were higher in responders (33.8 and 441.0 cells/mm^2^, respectively) than in nonresponders (17.3 and 142.0 cells/mm^2^, respectively), but this was not statistically significant (*P* = 0.84 and *P* = 0.14, respectively; Supplemental Figure 4A).

We performed gene set enrichment analysis (GSEA) of the MSigDB Hallmark gene set collection (32) on the 2,033 genes differentially expressed (unadjusted *P* < 0.05, 85 remained with adjusted *P* < 0.05) between responders and nonresponders (top 50 in Supplemental Figure 4B). We observed an enrichment of the MYC, MTORC1, and several immune-related signaling pathways in responders prior to ICI, including the IFN-γ, IFN-α, and TNF-α pathways (Figure 4D). The ultraviolet radiation (UVR) response pathway was also enriched in responders’ samples. Nonresponders demonstrated enrichment of the epithelial-mesenchymal transition (EMT) pathway, in agreement with findings in patients with CM not responding to neoadjuvant anti–PD-1 and anti–CTLA-4 ICI (21) (Figure 4D).

### Immunosuppressive cell populations and TGF-β signaling may contribute to ICI resistance in more-infiltrated MucM.

Patients with a MucM microenvironment that was classified as more-infiltrated based on RNA-Seq data (Figure 3D) responded more frequently to ICI (6 of 13, 46%) than patients with a less-infiltrated immune microenvironment (4 of 22, 18%), but this was not statistically significant (*P* = 0.12). Seeking to explore which resistance mechanisms might prevent an ICI response in these more-infiltrated MucM samples, we determined the TIDE (33) RNA signatures. These signatures reflect the presence of 3 suppressive immune populations (34–36): cancer-associated fibroblasts (CAFs), myeloid-derived suppressor cells (MDSCs), and M2 tumor-associated macrophages (TAMs; Figure 4E). We compared the 6 ICI-responding samples to the 7 nonresponding samples from the more-infiltrated cluster. While the signature for MDSCs was similar, the CAF and TAM signatures were higher in the nonresponding samples, but this difference was only statistically significant for the TAM signature (*P* = 0.14 and 0.035, Figure 4F). Furthermore, the more-infiltrated but nonresponding samples were characterized by higher, but not significant, levels of a TGF-β signature (*P* = 0.14, Figure 4G). Finally, mIF data (available for 4 responding and 4 nonresponding samples from the more-infiltrated cluster) revealed a significantly higher median stromal density of CD8^+^ T cells for responding samples compared with nonresponding samples (490.0 and 156 cells/mm^2^, respectively, *P* = 0.029, Figure 4H). In contrast, the intratumoral CD8^+^ density did not significantly differ (117.0 and 91.4 cells/mm^2^, respectively, *P* > 0.99, Supplemental Figure 4C). This suggests that, even in the context of a more-infiltrated tumor, a high density of CD8^+^ T cells at the MucM invasive margin is associated with ICI response — in line with previous findings in CM (20).

## Discussion

Here, we report on a retrospective, exploratory analysis of the MucM immune microenvironment using RNA-Seq and mIF analyses in 101 patients with MucM arising in different organ sites. To our knowledge, this is the largest MucM cohort that has undergone molecular characterization. Our data suggest that head & neck MucM is characterized by a stronger T cell infiltration and higher IFN-γ signature level as compared with those of MucM from other organ sites. Our data could further be interpreted as suggesting that the ICI response rate is higher for patients with MucM, with evidence of preexisting antitumor immune activity in the microenvironment, which would align with findings in other solid cancers, including CM (21, 37, 38). However, likely due to the low number of patients treated with ICI in this cohort, these findings were not statistically significant and should be considered hypothesis generating.

It is probable that our findings of a lack of tumor-infiltrating lymphocytes and the weak IFN-γ signature of MucM are associated with this tumor’s relative resistance to anti–PD-1 and anti–PD-1+anti–CTLA-4 as compared with CM tumors (12). Genomic investigations by other groups have demonstrated a considerably lower TMB in MucMs compared with CM tumors (2.3–2.7 mutations/Mb vs. 36.3–49.2 mutations/Mb, respectively) (16–18), which renders this tumor less immunogenic and likely contributes to the comparative immune hypoinfiltration we observed in MucM. Our findings in the subcohort of patients with MucM treated with ICI could be interpreted as showing that the microenvironment of ICI-responding patients is more densely infiltrated with immune cells and support the continued use of ICI in MucM. In line with this hypothesis, another report of a cohort of 124 patients with rare, noncutaneous melanoma (non-CM) subtypes treated with anti–PD-1 ICI, found that nonprogressing tumors had a higher T cell inflamed gene expression profile, though only a minority of this study’s samples were MucM (*n* = 44 [36%]; remaining samples were acral melanomas) (27). Further exploratory analyses of our data suggest that an ICI response is potentially hindered by the presence of inhibitory immune populations (TAMs and CAFs), soluble factors (TGF-β), and a lack of CD8^+^ T cells in the tumor-associated stroma, even in the context of a relatively more-infiltrated microenvironment.

We showed that MucM of the H&N region is characterized by stronger CD8^+^ T cell, cytotoxic cell, and IFN-γ signatures as compared with MucM of lower body sites. This could reflect a more potent immune reaction induced by the higher TMB previously described for H&N MucM compared with other organ sites (17, 18). The relatively more favorable immune profile of H&N MucM tumors would be expected to translate to a significantly superior ORR upon ICI, which we did not observe in our small cohort (36% in H&N vs. 25% for lower organ sites collectively, *P* = 0.69). In a recent retrospective study, Dimitriou et al. similarly describe a numerically, but not significantly higher, ORR in H&N MucM (40%) compared with AR (33%) or urogenital MucM (24%) among 197 patients treated with combined anti–CTLA-4+anti–PD-1 drugs and found no differences at all in the 331 patients with MucM treated with anti–PD-1 monotherapy (39). Prospective data are needed to establish if there is a difference in the efficacy of ICI between H&N MucM and MucM of lower organ sites.

A subgroup analysis of a recent phase III trial of nivolumab plus ipilimumab in 533 patients with unresectable melanoma showed a comparatively poor ORR and survival among the 32 enrolled patients with MucM (14). In another trial, adjuvant toripalimab (anti–PD-1) did not significantly improve survival compared with adjuvant high-dose IFN-α2b among patients with resectable MucM (40). Our analyses suggest that MucM, perhaps due to its low mutational neoantigenicity and immune infiltration, will indeed require therapies beyond anti–PD-1 monotherapy or dual anti–PD-1 + anti–CTLA-4 ICI. We would like to speculate that a combination of anti–PD-1 and anti–CTLA-4 drugs with local therapies, like STING agonists (41, 42), oncolytic virus talimogene laherparepvec (T-VEC) injection (43), or additional cytokine provision (44, 45), could be beneficial to induce more clinical responses in patients with MucM. Our exploratory data further suggest that immunosuppressive, tumor-associated M2 macrophages may contribute to ICI resistance even in MucM that is more immune infiltrated — potentially offering a targetable immune population (46). Cytotoxic chemotherapy, though not standard of care in stage IV patients, may still play a role in the treatment of MucM as an adjuvant therapy in patients with resectable disease (47, 48) — perhaps using Ki67 expression as a treatment stratification biomarker (49). As MucM belongs to the rare cancers, prospective clinical data are scarce. In this context, the 48% ORR observed after toripalimab (anti–PD-1) plus axitinib (anti-VEGF) (50) and the 45% ORR upon treatment with atezolizumab (anti–PD-L1) combined with bevacizumab (anti-VEGF) (51) in stage IV patients with MucM, are promising data. When given neoadjuvantly to patients with resectable MucM in a recent phase II trial (52), toripalimab plus axitinib led to an encouraging pathological response rate of 33%, underlining this regimen’s potential in MucM and warranting further investigation.

An inherent limitation of studying rare cancers is the low number of available patients and tumors, which — especially when the response to a certain treatment is modest —often leaves comparative results statistically insignificant. Our ICI-treated MucM cohort is indeed small and characterized by heterogeneity in terms of the ICI type and previous treatments — we are therefore aware of the risk of overinterpretation of these exploratory data and emphasize the need for more prospective data on (combination) ICI efficacy in MucM. Finally, the RNA-Seq data of our MucM cohort and the previously generated CM dataset are inevitably influenced by sequencing batch effects, though sequence protocols and equipment were similar. However, the consistency of our results after computational batch effect correction reflects a degree of robustness.

In summary, this report on the immune microenvironment of a large cohort of patients with MucM describes differential levels of lymphocyte infiltration and IFN-γ signature levels across MucM tumors from different primary organ sites, with the highest values in H&N MucM. However, compared with CM, MucM tumors are generally characterized by an unfavorable immune profile, likely contributing to the relatively modest efficacy of (dual) ICI observed in patients with MucM. Moving combination ICI to earlier disease stages (neoadjuvant therapies), selecting patients with a higher IFN-γ signature for dual ICI, or offering novel (triple) therapies might improve the ICI efficacy for patients with this rare cancer. In that way our analyses of MucM offer rationales for designing more biologically informed immunotherapeutic strategies for this rare disease.

## Methods

### Patient cohort and sample collection.

In this retrospective analysis, patients diagnosed with melanoma of the mucosal surface of the H&N (sites: sinonasal cavities or oral cavity), FUT (sites: vulva, vagina, and urethra), and MUT (sites: glans penis or urethra) as well as AR MucM (sites: anus or rectum) or GI MucM (sites: colon, stomach, and esophagus) and treated at the NKI and LUMC between 1997 and 2020 were included, if archived tumor material was available. MucM arising from the FUT, MUT, AR, and GI sites were grouped and considered as “lower-body-region” MucM and compared with MucMs of the H&N region, where appropriate. FFPE MucM material was obtained from the tissue archive and reviewed in-house. An experienced melanoma pathologist revised tumor specimens and confirmed the diagnosis of MucM. Clinical data were retrieved from medical records through chart review.

### Immune checkpoint inhibition treatment and response assessment.

Patients treated with at least 1 dose of anti–CTLA-4, anti-PD1, anti–PD-L1, or a combination thereof in any line of systemic therapy for recurrent or metastatic MucM were identified. All MucM material for sequencing was obtained before the start of ICI. The best OR at any time after ICI initiation was determined. A tumor’s response to treatment was assessed using CT or FDG-PET as part of the patient’s care. For this study, response was designated based on the Response Evaluation Criteria In Solid Tumors (RECIST, in case of CT) (53) or the European Organization for Research and Treatment of Cancer criteria (EORTC, in case of PET) (54) — retrospectively abstracted from the available radiology reports and treating physician’s notes. An OR to ICI was defined as a CR or PR. Patients with SD or PD as best response were considered nonresponders. Patients with a mixed response, in whom some lesions regressed while others progressed, were annotated separately within the PD group.

### RNA-Seq and data analysis.

RNA was extracted from annotated FFPE slides using the AllPrep DNA/RNA FFPE isolation kit (Qiagen, catalog 80234) in accordance with the manufacturer’s instructions. Strand-specific libraries were generated using the TruSeq RNA Exome Library Prep Kit (Illumina), and the pooled libraries were enriched for target regions using the probe Coding Exome Oligos set (CEX, 45 MB) according to standard procedures (Illumina, no. 1000000039582v01). After analysis on a 2100 Bioanalyzer (Agilent), the libraries were sequenced with 65 base pair single-end reads on a HiSeq2500 using V4 chemistry (Illumina).

FastQ files were mapped to the human reference genome (Homo.sapiens.GRCh38.v82) using STAR (55) (v2.6.1d). Count data were generated with HTSeqcount (56) (v0.11.0) and normalized using DESeq2 (31) (v1.30.1). Normalized gene expression data per dataset was centered by subtracting the row means and scaling by dividing the columns by the standard deviation. Deconvolution of RNA-Seq data was performed using immune sell signatures previously defined by Danaher (30), signatures for Tumor Immune Dysfunction and Exclusion (TIDE) (33), an IFN-γ signature (23), and a signature for TGF-β activity (57). GSEA on ranked genes by using the Signal2Noise metric was performed using the Hallmark Gene Set Collection (32) on the BROAD javaGSEA standalone version (http://www.broadinstitute.org/gsea/downloads.jsp) with 10,000 permutations (58, 59).

We compared our MucM data with an OpACIN plus OpACIN-neo (21, 29) dataset, generated from the pretreatment lymph node metastasis biopsies of 64 patients with stage III CM treated with neoadjuvant nivolumab (anti–PD-1) and ipilimumab (anti–CTLA-4) prior to surgery. We used the sva package (28) for identification and correction of batch effects between the two datasets. For the analysis of batch-uncorrected datasets, the MucM and OpACIN-neo datasets were normalized using DESeq2 normalization (31).

### mIF staining.

Staining was performed on sequential 3 μm slides (from the same FFPE MucM blocks used for RNA-Seq) using a Ventana Discovery Ultra automated stainer (Hoffman-La Roche), using the 50-slide Opal Polaris 7-Color Manual IHC Detection Kit (Akoya Biosciences, catalog NEL861001KT) according to the manufacturer’s instructions. The following antibodies and concentrations were used: anti-CD3 (clone SP7, Thermo Fisher Scientific, catalog RM-9107-S, 1:400 dilution), anti-CD8 (clone C8/144B, Agilent, catalog M7103, 1:100 dilution), anti-CD68 (clone KP1, catalog M0814, Agilent, 1:300 dilution), anti-FoxP3 (clone 236A/47, Abcam, catalog ab20034, 1:100 dilution), and anti-CD20 (clone L26, Agilent, catalog M0755, 1:500 dilution), anti-SOX10 (clone BC34, Biocare Medical, catalog ACI3099C, 1:20 dilution), and anti-MelanA (clone A103, Agilent, catalog M7196, 1:1600 dilution). Anti-SOX10 and anti-MelanA antibodies were coincubated. Slides were additionally incubated with OPAL dyes (1:40 or 1:50 dilution as appropriate) and DAPI (1:25 dilution in Reaction Buffer).

Stained whole slides were imaged using the Vectra Polaris automated imaging system (Akoya Biosciences). Scans were made with the MOTiF protocol. Using inForm Tissue Analysis Software (v. 2.5.0, Akoya Biosciences), the MOTiF images were unmixed into 8 channels (DAPI, OPAL480, OPAL520, OPAL570, OPAL620, OPAL690, OPAL780, and auto fluorescence), exported to a multilayered TIFF file, and fused with HALO software (v. 3.2.1851.229, Indica Labs).

### Analysis of multiplex-stained slides.

Please refer to Supplemental Figure 2 for an overview of the digital image analysis workflow. We first assessed the whole slides at low magnification (Supplemental Figure 2A) and manually excluded regions of necrosis and areas where excessive pigmentation, tissue folds, or liquid drops obscured marker expression. Tumor-adjacent melanoma in situ, if present, was also excluded.

Next, the tumor area was manually demarcated using the HALO software annotation tools, guided by the corresponding H&E-stained slide and SOX10/MelanA staining. Following the melanoma guidelines proposed by the International Immuno-Oncology Biomarkers Working Group (60), peritumoral stromal tissue within 500 μm was considered tumor-associated. Therefore, the tumoral annotation layer was concentrically expanded outward for 500 μm using HALO software (Supplemental Figure 2B). A unique random forest tissue classifier was trained for each slide to classify tissue regions into a “tumor” or “stroma” class (Supplemental Figure 2C). A cell segmentation algorithm was created for each slide based on the Indica Labs Highplex FL (v. 4.0.2) algorithm (Supplemental Figure 2D). Finally, the whole annotation layer (containing the whole tumor + 500 μm of peritumoral stroma) was analyzed, creating object files (containing individual cell marker positivity, coordinates, and tumor/stroma classification) and summary files (containing tissue surface areas and analysis settings) for each slide, which were exported in CSV format.

Phenotype calling was performed based on the object files. Only the immune cells that penetrated the tumor parenchyma (i.e., within the tumoral tissue class) were considered tumor infiltrating (61); all others were considered stromal. Cell phenotypes were quantified per tissue type and normalized for tissue class surface area in mm^2^.

### Statistics.

Statistical analyses were performed in RStudio Server (v. 1.4.1725) and R (v. 4.0.2). Survival was assessed using a Kaplan-Meier analysis. Where applicable, survival curves were compared with a log-rank test. Group medians were compared using Wilcoxon’s rank-sum test. Fractions were compared using Fisher’s exact test. All statistical tests performed in this study were 2 sided, and reported *P* values are exact. A *P* value of less than 0.05 was considered statistically significant. For the GSEA, gene sets with a nominal *P* value of less than 0.05 were considered significantly enriched. The correlations between the Danaher immune cell signatures and cell densities assessed per mIF were described using Spearman’s Rho — *P* values for these correlations were corrected for multiple comparisons using the Holm-Bonferroni method. Heatmap dendrograms were constructed based on Ward’s minimum variance method with squaring of the dissimilarities (Ward’s D^2^) of the Euclidean distance.

### Study approval.

The use of archived FFPE MucM tumor samples and the retrieval of data from individual medical records were performed under a protocol (IRBd18-095) approved by the NKI IRB. A data and material transfer agreement was signed between the NKI and LUMC. All procedures in this study were conducted pursuant to Dutch and international legislative and ethical standards. Prior to May 25, 2018, national legislation on data protection applied as well as the International Guideline on Good Clinical Practice. From May 25, 2018, onward we also adhere to the General Data Protection Regulation (GDPR). Within this framework, patients were informed and always had the opportunity to object or actively consent to the (continued) use of their personal data and biospecimens in research.

### Data availability.

All deidentified RNA-Seq data is available from European Genome-phenome Archive (EGAD50000000892; EGAS50000000631). Interested parties are asked to submit a data access request to the NKI Data Access Committee. Upon receipt and review of the data access request, interested parties will receive a Data Transfer Agreement to be completed, signed, and returned to the Data Access Committee, prior to being granted access to the requested dataset.

## Author contributions

JLV, CLZ, JBAGH, and CUB conceptualized and designed this research project. JLV, JJHT, MIEVP, and EK collected and curated the data. JVDW confirmed the diagnosis of mucosal melanoma and selected slides for RNA sequencing, with assistance from JLV. RNA sequencing and multiplex immunofluorescence experiments were carried out with assistance from IMS and DP. JJHT and JLV performed the bioinformatic analysis, statistical analyses, and visualization of the data. JLV, JJHT, and CLZ wrote the original manuscript draft. MWJMW, MBK, WMCK, AN, MVB, ORB, MIEVP, EK, CLZ, JVDW, JBAGH, and CUB were involved in the treatment of patients included in this study and provided clinical data. All authors, including XQ and LGTM, critically reviewed and edited the manuscript. Supervision was provided by CLZ, EH, AB, JBAGH, and CUB. Funding was acquired by JLV and CLZ.

## Supplementary Material

Supplemental data

Supporting data values

## Figures and Tables

**Table 1:**
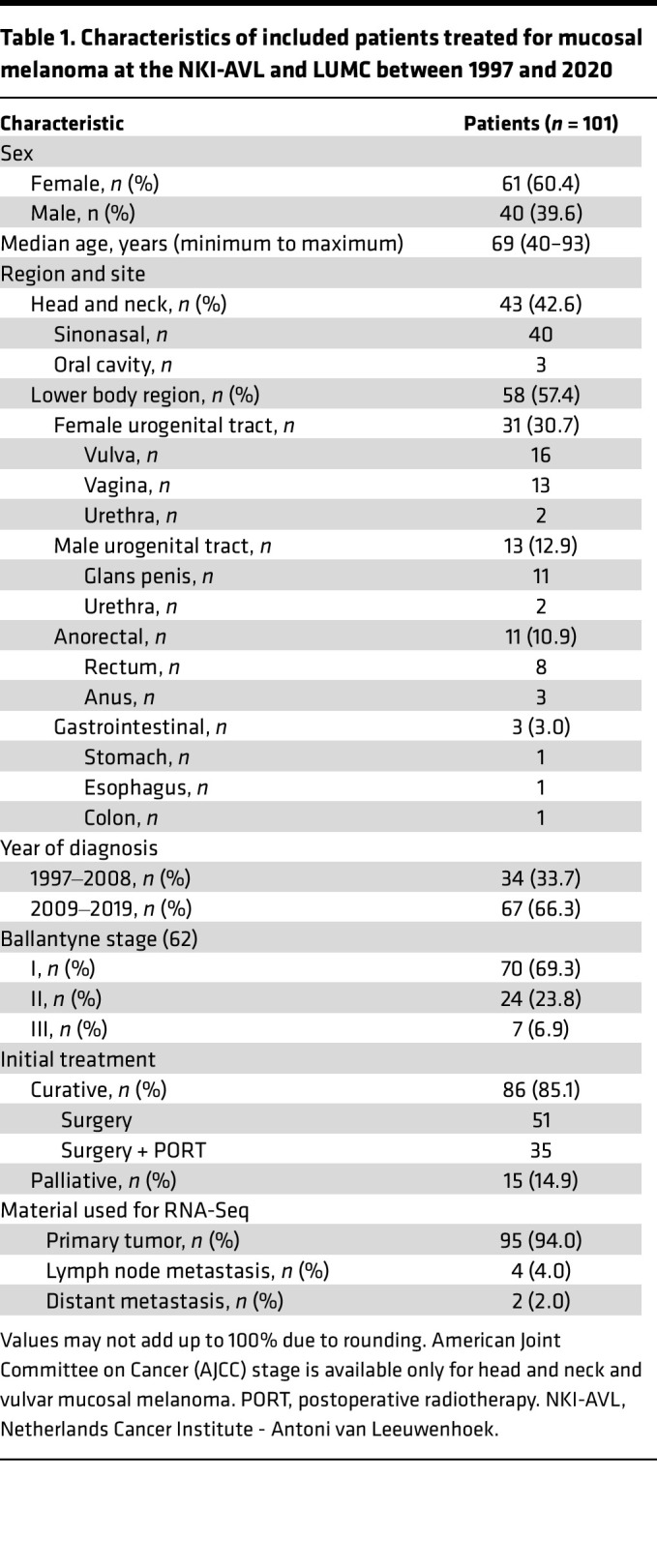
Characteristics of included patients treated for mucosal melanoma at the NKI-AVL and LUMC between 1997 and 2020

**Table 2:**
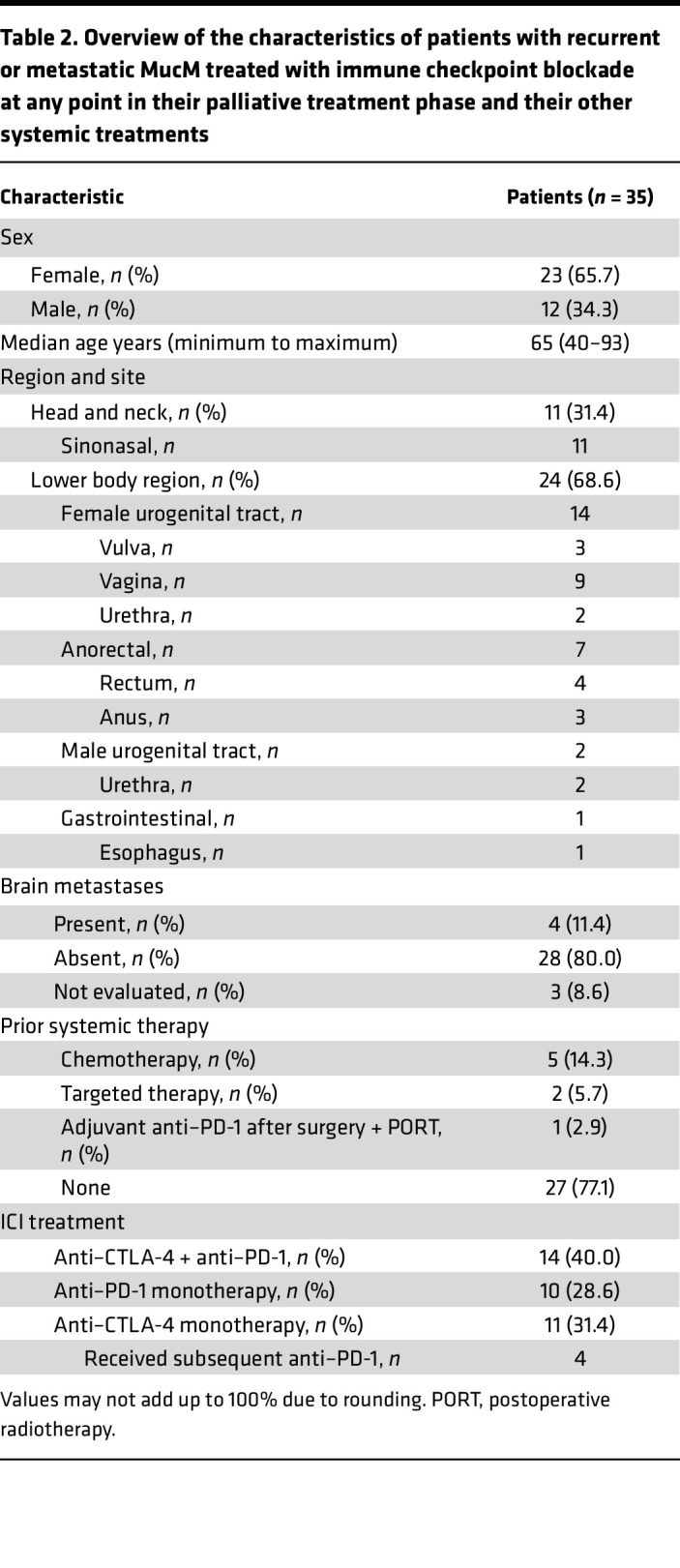
Overview of the characteristics of patients with recurrent or metastatic MucM treated with immune checkpoint blockade at any point in their palliative treatment phase and their other systemic treatments

**Table 3:**
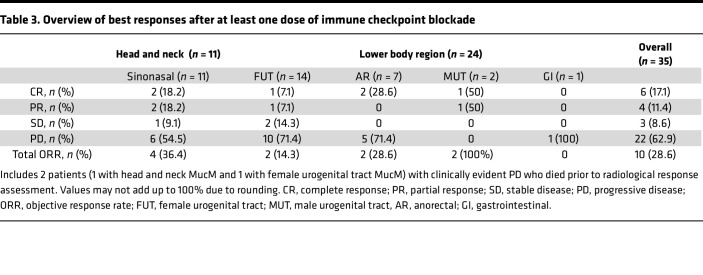
Overview of best responses after at least one dose of immune checkpoint blockade

## Version 1 11/08/2024

Electronic publication
