# Supplementary material for: Diversity of the immune microenvironment and response to checkpoint inhibitor immunotherapy in mucosal melanoma
Source: JCI Insight. 2024 Nov 8;9(21):e179982. doi: 10.1172/jci.insight.179982 (PMC11601749; doi:10.1172/jci.insight.179982)
Supplement: Supplemental data [file jciinsight-9-179982-s269.pdf]

SUPPLEMENTAL FIGURES

Supplemental Fig. 1

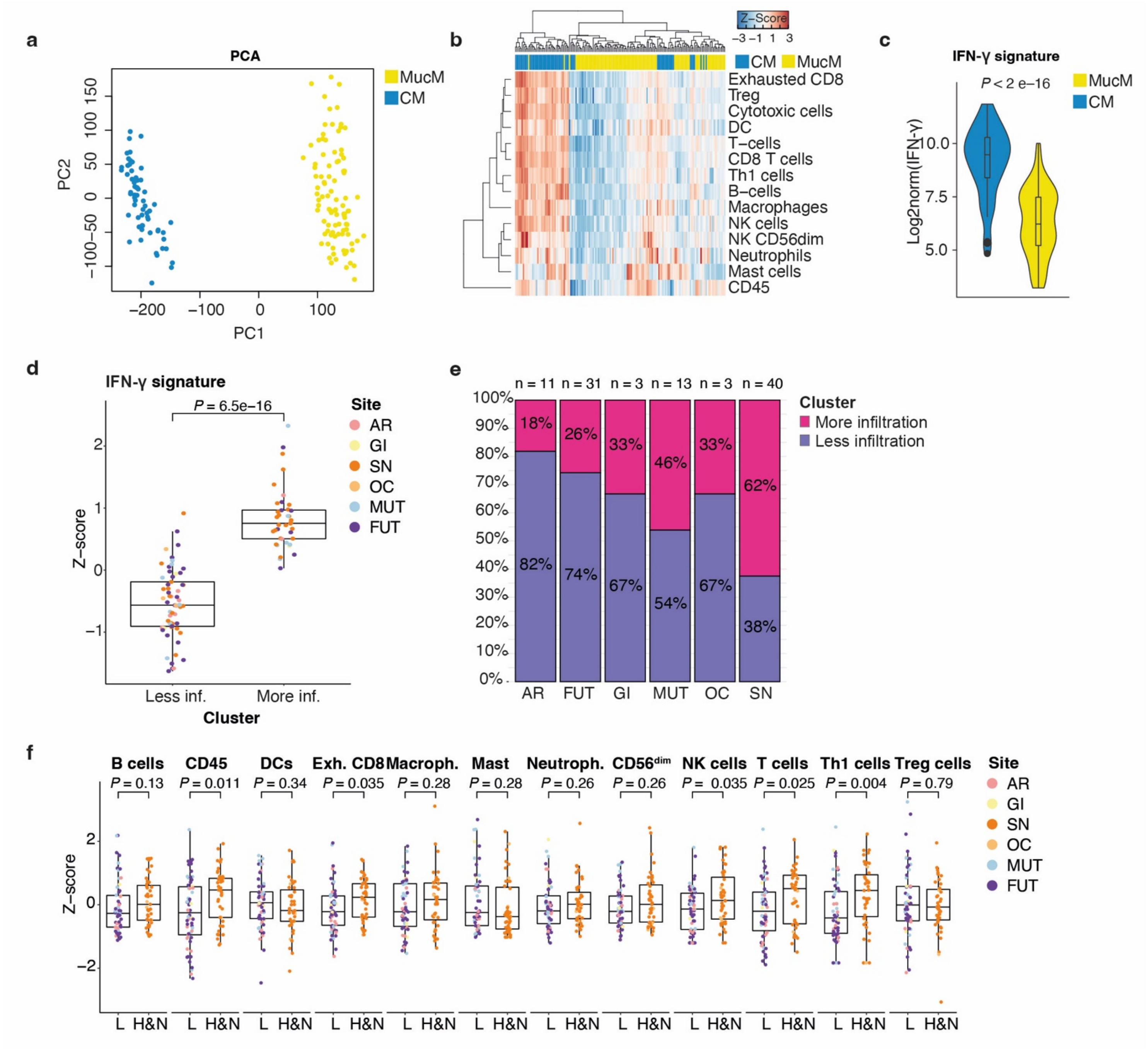

Supplemental Fig. 1 | Immune deconvolution of RNAseq data illustrating the MucM transcriptome in view of cutaneous melanoma (CM) and at different primary MucM sites

a, Principal component analyses (PCA) of all MucM and CM samples (DESeq2-normalized)[32]. Dot colors indicate if a sample is MucM or CM. **b**, Heat maps with hierarchical clustering of CM and MucM samples per the Danaher[31] immune cell signatures, normalized per DESeq2. Tracks show the tumor type per sample. **c,** Box-and-violin plots showing the 10-gene IFN-γ signature[21] in DESeq2-normalized MucM compared to CM samples. **d**, Box plot showing the IFN-γ signature[21] in samples classified as relatively less or more infiltrated according to hierarchical clustering of the Danaher[31] gene sets in **Fig. 3d**. **e**, Stacked bar plot showing the fraction of MucMs that clustered as more or less infiltrated in **Fig. 3d** per site of primary MucM origin. **f**, Box plots showing the Z-scores of all Danaher immune cell subsets (except cytotoxic and $CD8^+$ T cells, which are shown in **Fig. 3g**), in patients with H&N or lower region MucM.

The boxes show the median interquartile range (IQR); the whiskers extend from the minimal to the maximal value, but no further than 1.5x the IQR. The 'violin' displays the probability density of the data. The *P*-values are calculated using a two-sided Wilcoxon rank-sum test. MucM, mucosal melanoma; CM, cutaneous melanoma; PCA, principal component analysis; L, lower body region; H&N, head and neck; SN, sinonasal; OC, oral cavity, FUT, female urogenital tract; MUT, male urogenital tract; AR, anorectal; GI, gastro-intestinal.

22 **Supplemental Fig. 2**

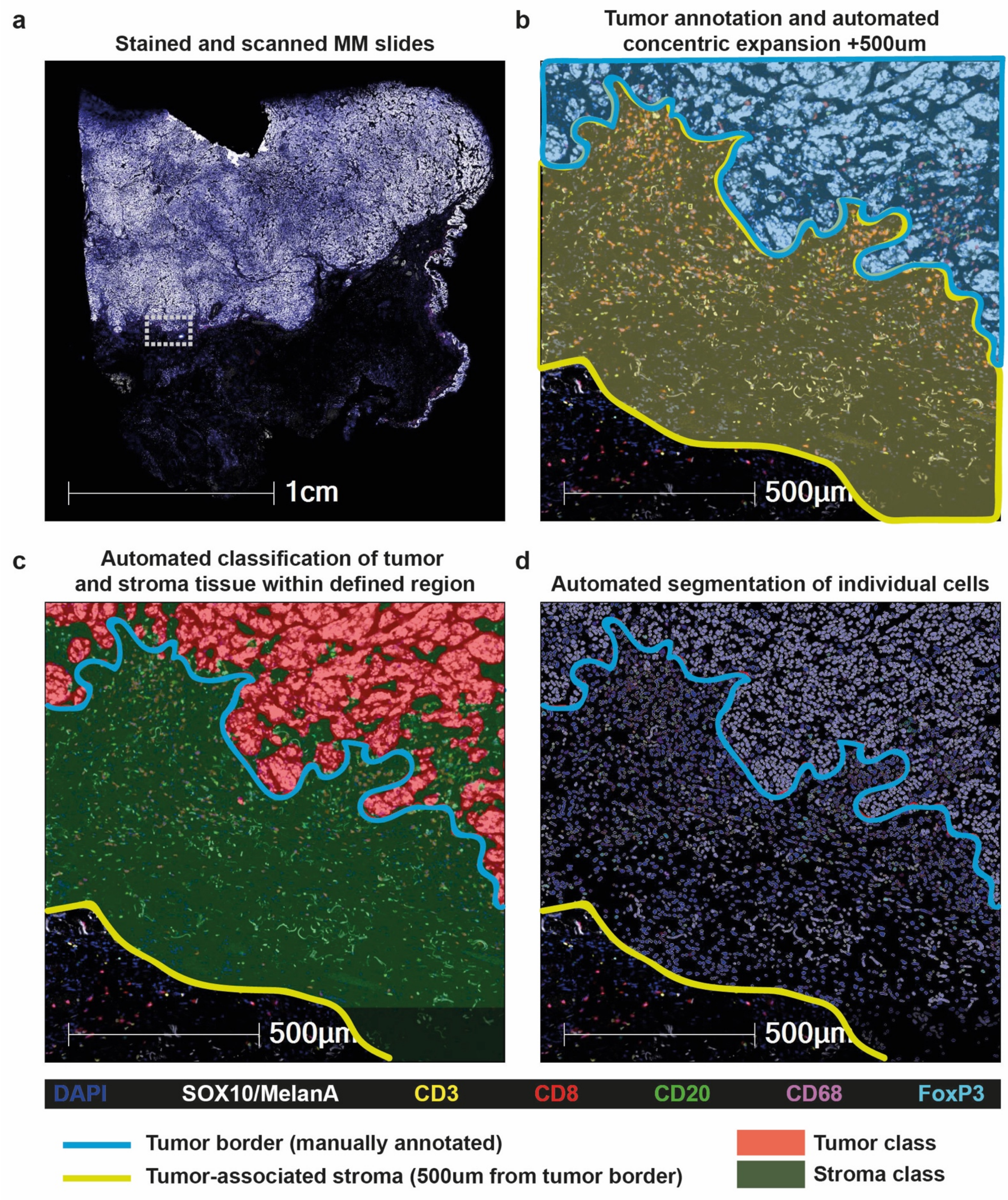

Supplemental Fig. 2 | Overview of the multiplex immunofluorescence (mIF) digital image analysis workflow

a, Low-magnification overview of a stained (markers and their respective colors are shown in the bottom track) and scanned primary MucM tumor; the dotted square is the region shown at higher magnification in panels **b**–**d**. **b**, Manual annotation of the invasive tumor (blue) on whole slides, and automated outward expansion of the tumor annotation by 500 μm (yellow), yielding a total annotation layer that includes tumor + 500 μm of tumor-associated stroma. **c**, Training of an individual random forest tissue classifier per slide, to classify tissue into a tumor (red) or stroma (green) class. **d**, Training of an individual algorithm to segment cells within the tumor and tumor-associated stroma. Object files containing individual cell coordinates, marker expression, and tumor / stroma classification are then exported for downstream analysis.

Supplemental Fig. 3

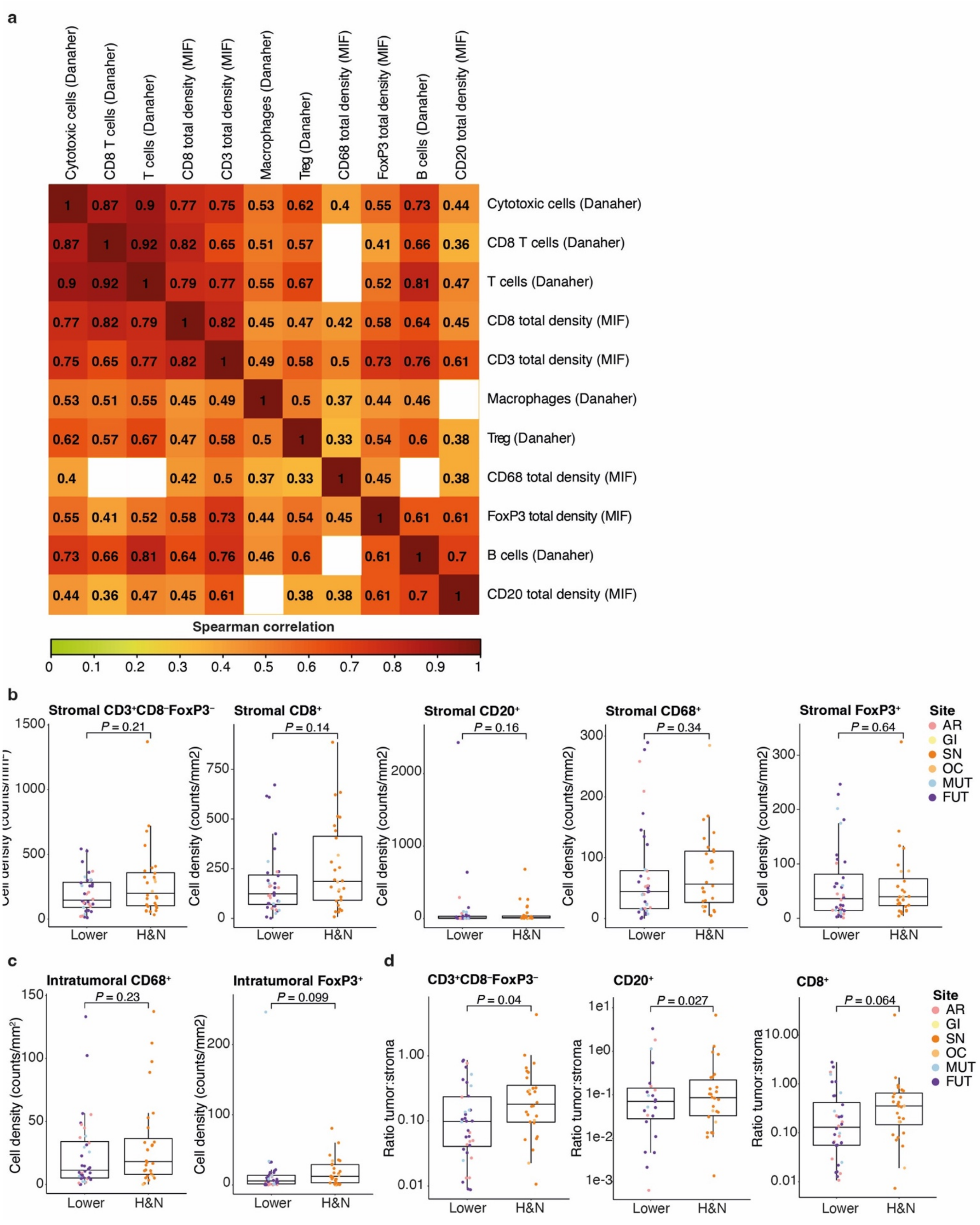

Supplemental Fig. 3 | Additional analyses of the MucM microenvironment at different sites using digital image analysis of multiplex-stained slides

a, Clustered correlation matrix showing the correlation between the Danaher gene expression signatures and the immunofluorescence-assessed, total cell density for the different immune phenotypes. The color intensity shows the Spearman correlation coefficient, which is also noted in each square. Only the correlations with a Holm-Bonferroni-corrected *P*-value < 0.01 are shown; insignificant correlations are left blank. **b**, Box plots showing the cell density in the tumor-associated stroma for $CD3^{+}CD8^{-}FoxP3^{-}$, $CD8^{+}$, $CD20^{+}$, $CD68^{+}$, and $FoxP3^{+}$ cells, in patients with primary H&N or lower region MucM. **c**, Box plots showing the intratumoral densities of $CD68^{+}$ and $FoxP3^{+}$ cells, in patients with primary H&N or lower region MucM. **d,** Box plots showing the intratumoral-to-stromal-ratio for the $CD3^{+}CD8^{-}FoxP3^{-}$, $CD20^{+}$, and $CD8^{+}$ cell densities, in patients with primary H&N or lower region MucM. Please note the log10-transformation of the Y-axes.

The box plots in panels **b–d** show the median + interquartile range (IQR); the whiskers extend from the minimal to the maximal value but no further than 1.5x the IQR. Individual dot colors represent the sample's primary site of origin. Exact *P*-values were calculated using a two-sided Wilcoxon rank-sum test. H&N, head and neck; SN, sinonasal; OC, oral cavity, FUT, female urogenital tract; MUT, male urogenital tract; AR, anorectal; GI, gastro-intestinal; mIF, multiplex immunofluorescence.

Supplemental Fig. 4

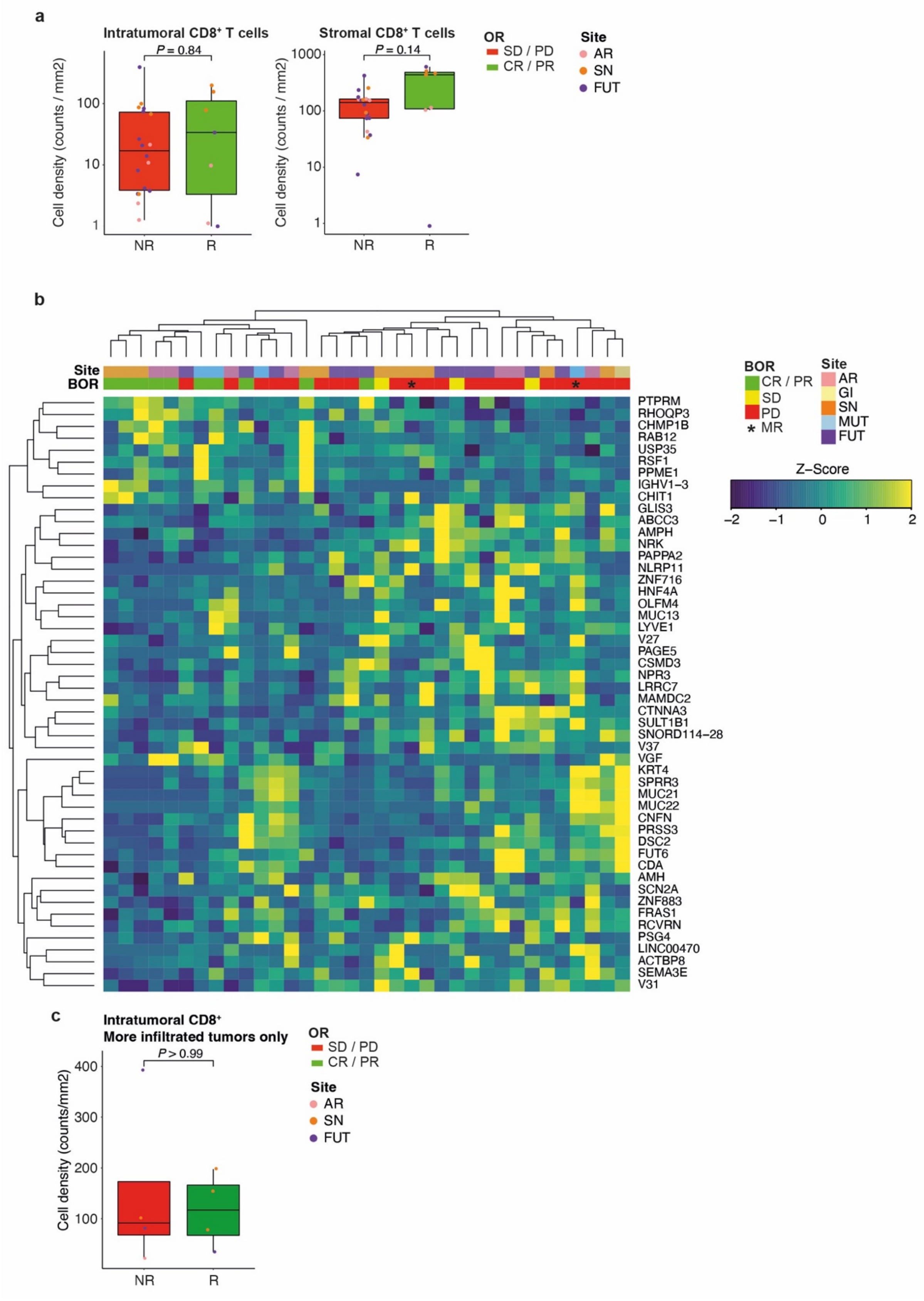

Supplemental Fig. 4 | Additional correlates of response to immune checkpoint blockade in ICI

a, Box plots visualizing the intratumoral (left) and stromal (right) density of $CD8^+$ T cells in patients responding (R, green) and non-responding (NR, red) to ICI treatment. **b**, The top 50 differentially expressed genes in responders versus non-responders. Tracks annotate a sample's primary site of origin and the patient's best objective response. Patients with a mixed response were considered having PD and are annotated with an asterisk. **c**, Box plots of the intratumoral $CD8^+$ T-cell density in responders (green) versus non-responders (red) in the more infiltrated cluster.

The box plots in panels **a** and **c** show the median + interquartile range (IQR); the whiskers extend from the minimal to the maximal value but no further than 1.5x the IQR. Individual dot colors represent the sample's primary site of origin. Exact *P*-values were calculated using a two-sided Wilcoxon rank-sum test. H&N, head and neck; SN, sinonasal; OC, oral cavity, FUT, female urogenital tract; MUT, male urogenital tract; AR, anorectal; GI, gastro-intestinal; CR, complete response; PR, partial response; SD, stable disease; PD, progressive disease; MR, mixed response; R, response; NR, no response.

SUPPLEMENTAL TABLES

Supplemental Table 1 | Immune cell density per tissue compartment and primary mucosal melanoma sublocation

Phenotype	All sites (N=64)	Sinonasal (N=25)	Oral cavity (N=3)	Anorectal (N=9)	FUT (N=21)	MUT (N=6)
**CD3$^+$CD8$^-$ FoxP3$^-$**						
Intratumoral	21.2 (6.0–59.0)	49.7 (20.4–102.9)	21.9 (14.4–53.7)	6.2 (4.7–16.0)	13.8 (4.9–38.1)	21.4 (13.0–33.8)
Stromal	179.1 (95.5–310.1)	173.4 (97.4–371.1)	288.4 (251.2–297.0)	124.7 (97.8–193.0)	195.4 (115.3–299.2)	173.5 (89.4–293.6)
**CD8$^+$**						
Intratumoral	27.9 (7.0–91.2)	86.8 (26.7–184.0)	53.4 (28.1–59.5)	10.8 (2.4–26.1)	13.9 (3.9–74.0)	17.8 (9.5–41.5)
Stromal	154.3 (73.7–262.7)	189.2 (87.8–441.3)	183.8 (164.1–250.9)	116.8 (104.0–160.2)	154.3 (73.9–235.3)	79.2 (48.6–153.1)
**CD20$^+$**						
Intratumoral	0.3 (0–3.0)	1.1 (0.3–6.9)	0.3 (0.2–0.4)	0.1 (0–0.2)	0.2 (0–1.7)	0 (0–3.8)
Stromal	11.8 (3.1–32.4)	14.7 (7.7–43.0)	7.7 (7.3–12.5)	2 .1 (1.0–12.0)	12.2 (3.2–32.0)	9.2 (1.8–69.1)
**CD68$^+$**						
Intratumoral	14.8 (6.2–34.6)	18.7 (10.9–45.0)	6.8 (3.4–17.2)	33.1 (4.7–44.9)	10.5 (5.4–22.7)	16.2 (3.3–28.6)
Stromal	49.8 (19.3–107.2)	53.6 (28.2–109.8)	82.1 (50.4–183.3)	53.7 (39.6–78.1)	44.4 (15.6–121.6)	18.9 (13.2–35.0)
**FoxP3$^+$**						
Intratumoral	8.6 (1.6–18.3)	12.1 (3.3–28.9)	13.8 (6.9–24.2)	1.4 (0.5–3.0)	8.1 (4.4–17.8)	10.8 (3.3–28.9)
Stromal	38.8 (15.0–77.4)	33.9 (21.4–58.6)	86.2 (63.0–86.5)	18.6 (10.5–42.5)	56.9 (17.4–103.6)	37.7 (12.7–146.2)

Medians and respective interquartile ranges are presented. FUT, female urogenital tract; MUT, male urogenital tract; Q1, first quartile; Q3, third quartile.
